# Analysis of Infection Time Courses Shows CII Levels Determine the Frequency of Lysogeny in Phage 186

**DOI:** 10.3390/ph14100998

**Published:** 2021-09-29

**Authors:** Nan Hao, Dylan Agnew, Sandeep Krishna, Ian B. Dodd, Keith E. Shearwin

**Affiliations:** 1 Department of Molecular and Biomedical Science, School of Biological Sciences, The University of Adelaide, Adelaide, SA 5005, Australia; nan.hao@adelaide.edu.au (N.H.); Dylan.Agnew185@schools.sa.edu.au (D.A.); ian.dodd@adelaide.edu.au (I.B.D.); 2 CSIRO Synthetic Biology Future Science Platform, CSIRO, Canberra, ACT 2601, Australia; 3 Simons Centre for the Study of Living Machines, National Centre for Biological Sciences TIFR, GKVK Campus, Bellary Road, Bangalore 560065, India; sandeep@ncbs.res.in

**Keywords:** lysogeny, phage infection, multiplicity of infection, bacteriophage, temperate phage, phage therapy, CII protein, synthetic biology

## Abstract

Engineered phage with properties optimised for the treatment of bacterial infections hold great promise, but require careful characterisation by a number of approaches. Phage–bacteria infection time courses, where populations of bacteriophage and bacteria are mixed and followed over many infection cycles, can be used to deduce properties of phage infection at the individual cell level. Here, we apply this approach to analysis of infection of *Escherichia coli* by the temperate bacteriophage 186 and explore which properties of the infection process can be reliably inferred. By applying established modelling methods to such data, we extract the frequency at which phage 186 chooses the lysogenic pathway after infection, and show that lysogenisation increases in a graded manner with increased expression of the lysogenic establishment factor CII. The data also suggest that, like phage λ, the rate of lysogeny of phage 186 increases with multiple infections.

## 1. Introduction

The ability of temperate bacteriophage to choose between two alternative lifestyles, lysis and lysogeny, provides simple systems for examining how genetic circuits can encode decision making, signal responsiveness and developmental commitment.

In phage λ, the best understood of these systems, lysogenic or lytic development and the signal-regulated transitions between them are controlled by a small network of regulatory proteins [1,2,3,4,5,6]. Commitment to either lysogeny or lysis, respectively, is provided by the lysogenic repressor CI or the late gene antiterminator Q. The choice between lysis and lysogeny after infection is generally thought to be determined by the cellular level of the CII protein. CII is required for establishment of lysogeny; it is necessary for effective expression of CI (and the integrase), and it also inhibits expression of Q [7,8,9]. CII is sensitive to host proteases [10,11], and environmental factors that influence the lysis-lysogeny decision, such as the multiplicity of infection (MOI), nutritional status, temperature, and cell size [12,13,14], are thought to act by affecting the rate of this proteolysis [8].

**Figure 1:**
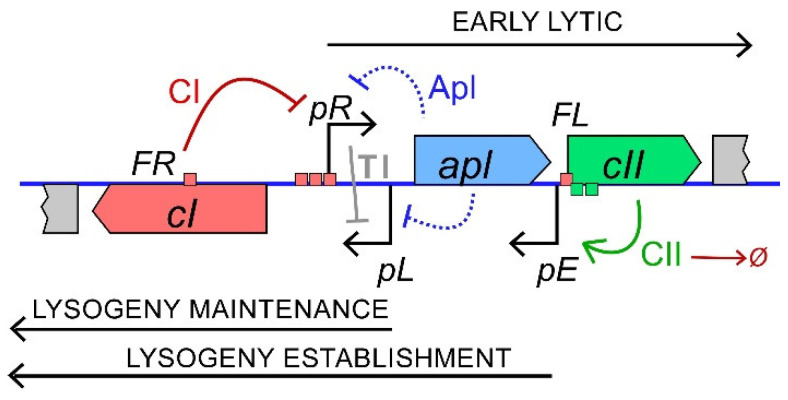
The lytic-lysogenic switch region of bacteriophage 186. Establishment of lysogeny requires the following steps. After infection, *pR* is on and represses lysogenic transcription by transcriptional interference (TI) with the weak *pL* promoter. Transcription from *pR* leads to the production of CII and Apl. CII activates *pE* to produce CI, while Apl cooperatively represses both *pR* and *pL* (dashed lines). If sufficient CI is produced, *pR* is repressed, lytic development is halted, and the phage enters lysogeny. The transcriptional interference (TI) from *pR* at *pL* is alleviated, allowing *pL* to maintain CI production. CII is subject to degradation by host proteases. The bent arrows represent promoters and the dashed lines represent repression by Apl. The red and green boxes indicate the CI and CII binding sites, respectively.

We have been studying the lysis-lysogeny system of the P2 family temperate coliphage 186 as an alternative simple decision-making genetic circuit [14,15,16]. Phage 186 expresses a protein, also called CII, which is essential for establishment of lysogeny and regulates gene expression in a similar manner to the unrelated λ CII protein. 186 CII is produced from the early lytic *pR* transcript and activates transcription of the lysogenic operon from the *pE* promoter to express the CI lysogenic repressor and the 186 integrase [17,18] (Figure 1). 186 CI enforces lysogeny by repressing the early lytic promoters, including *pR*, and activating its own promoter, *pL* [15]. CI also represses the promoter for the late gene activator B [19]. 186 CII is rapidly degraded by the host RseP and FtsH proteases in vivo [17]. We hypothesised that, like λ, the cellular concentration of 186 CII is the critical factor in its lytic → lysogenic decision. Consistent with this, a 186 phage expressing a stabilised version of CII has a frequency of lysogeny (FOL) close to 100% [17].

To examine whether the cellular concentration of CII determines the FOL for phage 186, we analysed infection time courses to estimate the FOL of cII-deficient 186 phage in response to different fixed CII levels. Infection time courses involve mixing phage with an excess of bacteria and following the optical density of the culture over time. For phage that cause cell lysis, this allows the progress of the infection of the bacterial population to be followed. The low multiplicity of addition (MOA) of phage means that multiple rounds of infection occur before major effects on the culture are seen. Time course data can provide information about infectivity, rates of phage production and the FOL, with large amounts of data able to be readily obtained using microtitre plates [20]. By optimising the fitting of such data to predator-prey infection models, we were able to quantitate key infection parameters. We show that the 186 FOL increases in a gradual manner as the cellular level of CII is increased. In addition, our analysis suggests that the phage 186 lysis-lysogeny decision responds to the multiplicity of infection.

## 2. Results

### 2.1. Analysis of Phage 186 Infection Time Courses with a Simple Lysogenisation Model

**Figure 2:**
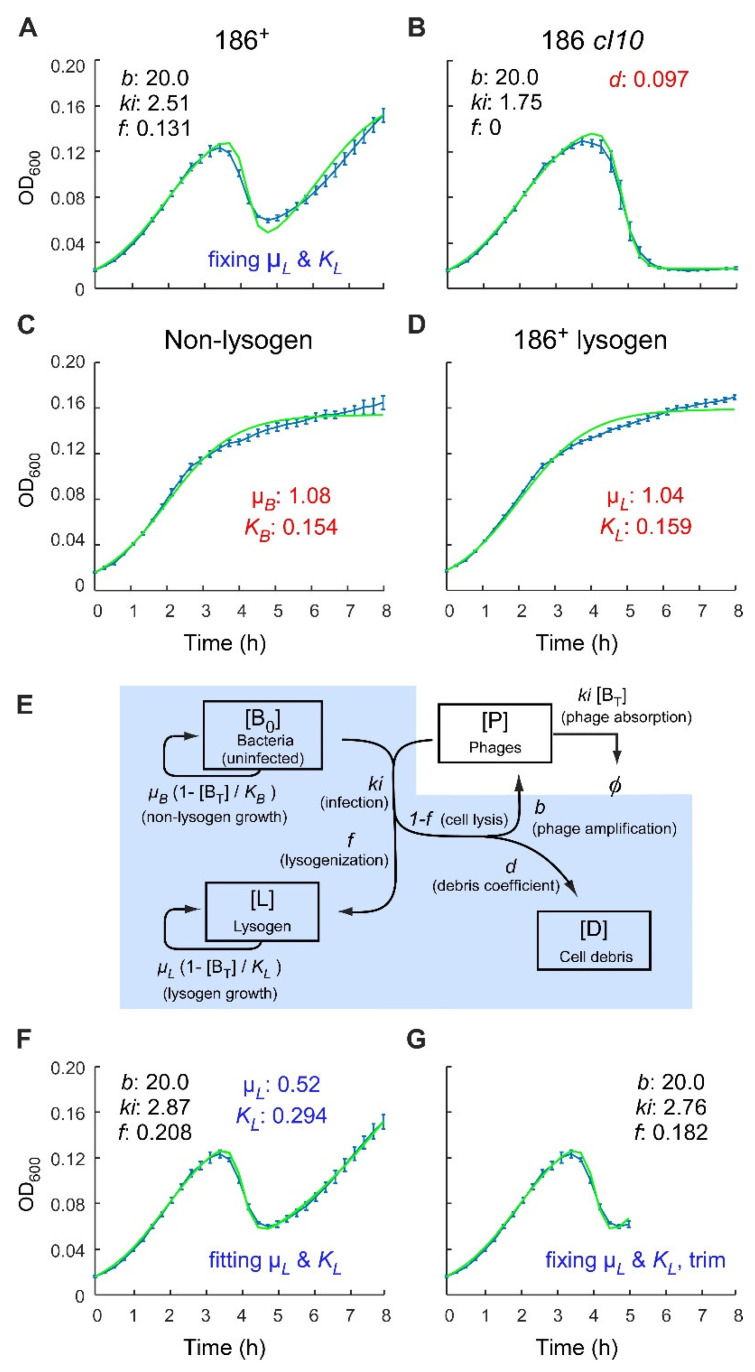
Analysis of 186 phage infection time courses with a simple lysogenisation model. (**A**) Infection time course data (blue) and fit (green) following 186^+^ (wild type) phage infection. (**B**) The cell debris coefficient *d* was obtained by fitting 186 cI10 infection curve by setting lysogenisation frequency (f) to 0. The growth parameters (µ_B_, K_B_, µ_L_, and K_L_) were fixed at the best fit parameters from fitting the nonlysogen (**C**) and 186^+^ lysogen (**D**) growth curves. (**E**) Schematic representation of the assumptions underlying the lysogenisation model. Populations that contribute to OD_600_ readings are shaded in blue. (**F**) A better fit to the infection data was obtained when the lysogenic growth parameters were allowed to vary. In this case, the best fit lysogen growth rate was ~1/2 that from the growth curve fit (panel **D**). (**G**) A good fit to the wild type 186 infection curve was also obtained with predetermined growth parameters (panels **C**,**D**) when the infection curve is trimmed to two points beyond the trough minimum (~5 h). All infections were performed at MOA of 1.5 × 10^−4^. Error bars represent standard deviation, *n* = 6. For data fitting, three rounds of 50,000 iterations of fitting were performed, and the best fit from each round was plotted.

Infection time courses were obtained by the method of Maynard et al. [20], in 96-well microtitre plates maintained with shaking at 37 °C in a spectrophotometer. Wild type 186 (186^+^) phage was added to a log phase culture of BW25113 cells at 3.1 × 10^6^ cells per well in rich medium, at an initial phage to cell ratio (MOA) of 1.5 × 10^−4^. The OD_600_ of infected cultures was followed for 8 h (Figure 2A). Temperate phage infection generally follows three distinct phases. At low MOAs, the number of phage at the initial stage of infection is insignificant in comparison to cells, so that the cell population initially grows exponentially with minimum influence from the phage, resulting in increasing OD_600_ that is similar to an uninfected culture. However, the phage reproduce at a faster rate than the bacteria, so eventually a large fraction of cells becomes infected and lyses, resulting in a drop in OD_600_. After this peak, the growth of immune lysogens leads to the recovery of the OD_600_. This postlysis outgrowth was absent after infection by 186 *cI10*, a clear plaque mutant that cannot lysogenise [21] (Figure 2B). The depth of the postlysis ‘trough’ thus gives an indication of the frequency of lysogeny. Uninfected cultures (Figure 2C,D) show no such trough in the infection curve.

We attempted to quantitate the lysogenisation frequency of phage 186 from this data by using a simple infection model modified from Maynard et al. [20]. Let B_T_ = B_0_ + L.

d[B_0_]/dt = *μ_B_* [B_0_] (1 − [B_T_]/K_B_) − *ki* [B_0_] [P] (1)

d[P]/dt = *b ki* (1 − *f*) [B_0_] [P] − *ki* [B_T_] [P] (2)

d[L]/dt = *μ_L_* [L] (1 − [B_T_]/K_L_) + *ki f* [B_0_] [P] (3)

d[D]/dt = *d ki* (1 − *f*) [B_0_] [P] (4)

The Maynard model considers three populations (Equations (1)–(3)), uninfected bacteria (B_0_), lysogens (L), and phage (P) (Figure 2E). The concentrations of the bacterial species [B_0_ + L] are measured by OD_600_, which can be considered as proportional to the number of cells per unit volume. The units for the phage concentration are the same, even though the phage are too small to scatter any measurable amount of light at 600 nm. We modified the Maynard model by adding a cell debris species (D; Equation (4)), as it was found that even after complete cell lysis with 186 *cI10*, the cell lysate can scatter a substantial amount of light at 600 nm (Figure 2B), resulting in significantly higher OD_600_ readings than medium alone. D is produced by phage lysis and has the same concentration units as B_0_ and L, with all three of them contributing to the overall OD_600_ measurement.

The growth of bacteria and phage are modelled according to classical predator-prey dynamics [22]. The growth of bacteria is assumed to follow a logistic fashion at rate *µ* towards a carrying capacity K, the maximal total bacteria density that the system can sustain (Equations (1) and (3); Figure 2E). These parameters for the nonlysogen and lysogen were estimated by fitting growth curves for uninfected BW25113 and BW25113(186^+^) lysogen (Figure 2C,D). The best-fit parameters for growth rate *µ* and carrying capacity K for the nonlysogen strain were 1.09 h^−1^ and 0.15, and those for the lysogen were 1.04 h^−1^ and 0.16, indicating that both strains have similar growth profiles, with estimated doubling times of 38–40 min (= ln2/*µ*).

The rate of phage infection of nonlysogenic cells is dependent on the product of the concentrations of the phage [P], the host [B_0_], and the infection rate constant *ki* [hour^−1^ OD_600_^−1^], given by *ki* [B_0_] [P]. There are two possible outcomes following a temperate phage infection: (1) lysis of bacterial cells to release more phage, and (2) formation of lysis resistant lysogens. We define the frequency at which the infected cell undertakes the lysogenic pathway as *f*, and the lytic fraction is thus given by 1 − *f* (note that this is the reverse nomenclature of that used by Maynard et al. [20]).

At any given time, the change in phage concentration is given by the production of phage minus their loss due to absorption (Equation (2)). Phage loss occurs through absorption by both nonlysogens and lysogens (with rates *ki* [B_T_] [P]) and is lysogenisation-frequency-independent. Phage production is a product of the rate of infection of nonlysogens (*ki* [B_0_] [P]), the proportion of the infected cells that undergo lytic development (1 − *f*), and the unitless ‘burst’ coefficient *b*. Here, *b* is effectively a productivity factor that scales the phage ‘return’ relative to each phage ‘spent’ in infection of nonlysogens.

We found that the model does not allow strong specification of *ki* and *b* from the data, with a range of *ki* and *b* values giving similar fits. The estimates obtained for *ki* and *b* are strongly anticorrelated, presumably because phage production depends on their product (*ki.b*). Because cell lysis and phage production occur immediately upon infection in the model, *ki* must represent both a rate of phage removal by infection, and what is in reality a considerably slower rate of infection followed by phage production. We therefore do not attach particular significance to the estimates for *ki* and *b*. To simplify the fitting, we therefore constrained *ki* and *b* to phage-like values: a 20–50 min average time for infection and lysis (1.2 ≤ *ki* ≤ 3), and between a 20 and 200 phage return per single infecting phage for *b*.

The lysis of host cells results in the formation of cell debris, with a rate *d ki* (1 − *f*) [B_0_] [P] (Equation (4)). It is expected that the amount of light scattered by the cell debris may differ considerably from an intact cell. Thus, an additional coefficient *d* is introduced, which denotes the proportion of OD_600_ that the debris from a lysed cell contributes compared to a viable bacterial cell. By fitting the 186 *cI10* data (Figure 2B), we estimated *d* = 0.1 (Materials and Methods).

Finally, infection by temperate bacteriophage also gives rise to lysogens (Equation (3)). The rate of change in lysogen numbers over time can be expressed as a product of the lysogenisation frequency, the infection rate constant, and the concentrations of both nonlysogens and the phage, *ki f* [B_0_] [P].

The fit between the phage 186^+^ time course and this model was not entirely satisfactory (Figure 2A). In particular, the rate of increase of OD_600_ postlysis was substantially slower than that expected based on the growth rate of lysogens measured in the absence of infection (Figure 2D). Accordingly, allowing lysogenic growth parameters to vary in the fitting gave a lysogen growth rate ~1/2 that of the nonlysogen (Figure 2F). A slow growth rate in this phase might be explained by depletion of small molecule nutrients due to previous production of bacterial and phage mass. Another possibility is that lysogen growth may be inhibited by repeated phage infection. Whatever the cause, we decided to focus the fitting on those features that are determined by the frequency of lysogeny, the peak and the postlysis trough. We thus included only two points beyond the trough minimum in the fitting, giving an improved match of the model with the positions of the peak and trough after phage 186^+^ infection (Figure 2G).

Using this model and the trimmed data, the FOL, *f*, for phage 186^+^ was estimated to be ~18%. This value is slightly higher than the ~10% previously obtained from single round phage plating experiments performed at low MOAs [17]. However, it should be emphasised that the infection time course experiment involves multiple rounds of infection, and thus *f* reflects an *aggregate* lysogenisation frequency from multiple rounds of infection under changing conditions.

### 2.2. Control of the Frequency of Lysogeny by 186 CII

Extremes of CII activity are known to have strong effects on the frequency of lysogeny in phage 186. In the absence of active CII, 186 establishes lysogeny only rarely [17,21]. In contrast, high level CII expression due to a stabilising mutation in *cII* results in ~100% lysogeny [17]. If CII is a determining factor in the frequency of lysogeny, we would expect that intermediate CII activities should result in frequencies of lysogeny between these extremes.

We first tested the mildest of available phage mutants that are defective in CII activity. 186 KS54 is one of a set of *pE* mutants isolated from a genetic screen for phage that were able to form plaques on a strain constitutively expressing a high level of CII [18]. The KS54 mutation is an A to G change in the promoter-distal half-site of the CII binding site at *pE* (Figure 1) and retains substantial *pE* activity, reducing activation by CII to ~63% that of wild type *pE* at high levels of CII [18].

**Figure 3:**
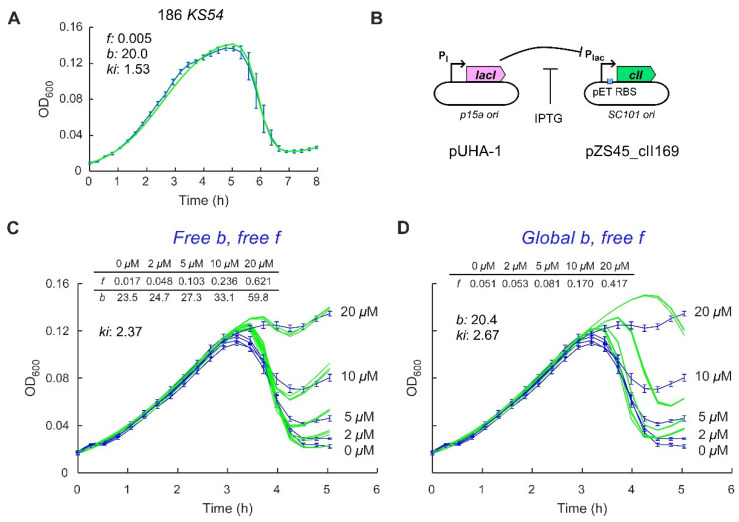
The role of CII in controlling the FOL in phage 186. (**A**) Infection time course (blue) and fittings (green) following 186 KS54 phage infection. (**B**) A two-plasmid system for controlled CII expression. The CII was expressed from low copy number pZS45_cII169 and was controlled by IPTG. The Lac repressor was expressed from a medium copy number plasmid (pUHA-1), under the control of its native promoter. (**C**,**D**) Infection time courses and fittings following 186 *cII*^−^ phage infection at five different IPTG concentrations. The fitting was performed with either free *b* for each IPTG concentration (**C**) or a global *b* (**D**). All infections were performed at MOA of 1.5 × 10^−4^. Error bars represent standard deviation, *n* = 6. For data fitting, three rounds of 50,000 iterations of fitting were performed, and the best fit from each round was plotted.

The growth rate of the 186 KS54 lysogen (~35 min per doubling, Figure S1A) was similar to the 186^+^ lysogen and the nonlysogenic parental strain, although the fitted carrying capacity K_L_ for the 186 KS54 lysogen was slightly lower. Infection by 186 KS54 produced a time course similar to that seen for 186 cI10, with model fitting giving a very low FOL of ~0.005 (Figure 3A). Thus, even a relatively mild defect in CII activity produces a strong decrease in the FOL, though it should be noted that we do not know how much the KS54 mutation affects *pE* activity at physiological CII levels.

To better test the effect of a range of CII levels on lysogenisation frequencies, we expressed CII under IPTG control from a plasmid and infected the cells with a 186 *cII*^−^ phage. Plasmid pZS45-186CII169 carries the wild-type *cII* gene downstream of the *placUV5* promoter that is repressed by LacI produced from a separate pUHA-1 plasmid (Methods; Figure 3B). Addition of IPTG leads to dose-dependent induction of CII, as judged by *pE* activity [23]. BW25113 carrying these plasmids was infected with 186 *cIIHTH*^−^, which carries mutations in the CII helix-turn-helix DNA binding motif that fully inactivate CII [23]. Addition of IPTG up to 20 µM had little effect on the growth of the nonlysogenic host strain or 186 *cIIHTH*^−^ lysogens of this strain, with the doubling time for both strains remaining at ~40 min, regardless of whether IPTG was added (Figure S1B–D). In addition, infecting CII-expressing cells with 186 *cI10* phage again resulted in complete cell lysis (Figure S1E).

In the infection time-courses with these phage and bacterial strains (Figure 3C), the depth of the postlysis trough became shallower with increasing CII expression, indicative of increasing FOLs. To fit these data with the model, we treated the five curves as a group. The bacterial growth parameters and *d* were fixed at the predetermined values and though *ki* was allowed to vary, its value was global, that is, shared for all curves. Individual values for *f* were allowed for the different IPTG concentrations. We also allowed individual values for *b*, as we thought that higher levels of CII might inhibit lytic development, as seen for λ [24,25]. A good fit between the data and the model was obtained with *f* estimates of 0.02, 0.05, 0.10, 0.24 and 0.62 for 0, 2, 5, 10 and 20 µM IPTG, respectively (Figure 3C). This gradually increasing FOL supports the idea that CII levels are a critical quantitative determinant in the phage 186 lysis-lysogeny decision. However, we were surprised that the estimates for *b* also increased with increasing CII expression. Fitting of the five time-courses in which the fitted value for *b* was applied globally gave poor fits (Figure 3D). It does not make sense to us that a lysogeny-promoting factor should make lytic development, when it is chosen, *more* efficient.

We reasoned that the increase in *b* is a way for the model to compensate for decreased phage production in the prepeak phase due to increased frequency of lysogeny. A lower rate of phage production in this early phase causes a later timing of the peak, because the peak occurs when the phage numbers have accumulated to the point at which the number of bacteria lost through infection equals the number of bacteria gained by cell division. All else being equal, a higher *f* should thus tend to delay the peak, as seen in the fits where *b* is global (Figure 3D). Since the observed peak times are similar, at least up to 10 µM IPTG, and the *f* values are primarily determined by the depth of the postpeak trough, the model can only fit the data by increasing *b* as *f* increases.

A possible solution to this conundrum is if lysogenisation in the prepeak phase is at a relatively low frequency and is thus less deleterious to phage production, while the frequency of lysogenisation in the later phase is higher and able to rapidly produce the level of lysogens observed by the trough depth. One mechanism that could achieve this is if the phage 186 lysis-lysogeny decision is MOI sensitive, with the FOL for an individual cell increasing when the cell is simultaneously infected by more than one phage, as seen for λ [11,12,26]. Thus, in the prepeak period of the time course, low phage numbers would mean that MOIs rarely exceed 1 and the FOL would be low, allowing phage production to be relatively uninhibited by lysogenisation. At the peak and postpeak phase, phage numbers should exceed the number of bacteria, resulting in higher MOIs, higher FOLs and thus rapid accumulation of lysogens. To test this idea, we utilised a phage infection model developed by Sinha et al. [27] that allows different FOLs for multiple phage infections.

### 2.3. Application of a Multiple-Infection Lysogenisation Model to the CII Expression Time-Courses

**Figure 4:**
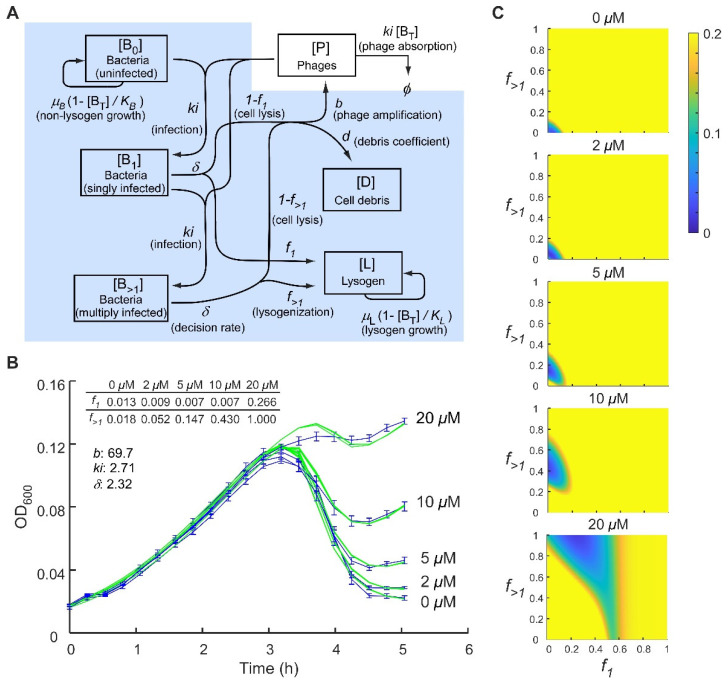
The multiple-infection lysogenisation model allows a better fit to the 186 *cII^−^* phage infection curves with a global *b*. (**A**) Schematic representation of the assumptions underlying the multiple-infection lysogenisation model. Populations that contribute to OD_600_ readings are shaded in blue. (**B**) Fitting of the 186 *cII*^−^ phage infection time courses at five different IPTG concentrations using the multiple-infection lysogenisation model. Data are taken from Figure 3C. Error bars represent standard deviation, *n* = 6. For data fitting, three rounds of 50,000 iterations of fitting were performed, and the best fit from each round was plotted. (**C**) Heatmaps showing the *f*_1_, *f*_>1_ value ranges that produce a good fit at different CII levels. Low scores (blue) indicate better fits.

The multiple-infection model (Figure 4A) introduces two new species, bacteria infected with one phage (B_1_) or with multiple phage (B_>1_), and a new rate *δ* that defines the timing of the lysis-lysogeny decision. Thus, singly infected bacteria (B_1_) make a decision at a rate *δ* either to become lysogenic (with probability *f*_1_) or to lyse and produce phage (with probability 1 − *f*_1_). Before this decision is made, they can be infected with a second phage to become multiply infected (B_>1_). These B_>1_ bacteria make the same lysis-lysogeny decision but with a different FOL (*f*_>1_). The B_>1_ cells can be further infected by phage, but this does not affect their decision and they remain as B_>1_. The growth of B_1_ and B_>1_ cells before lysis or lysogeny is assumed to be negligible, as the decision is fast relative to cell division. Let B_T_ = B_0_ + B_1_ + B_>1_ + L.

d[B_0_]/dt = *μ_B_* [B_0_] (1 − [B_T_]/K_B_) − *ki* [B_0_] [P] (5)

d[B_1_]/dt = *ki* [B_0_] [P] − *ki* [B_1_][P] − *δ* [B_1_] (6)

d[B_>1_]/dt = *ki* [B_1_] [P] − *δ* [B_>1_] (7)

d[P]/dt = *b δ* (1 − *f*_1_) [B_1_] + *b δ* (1 − *f*_>1_) [B_>1_] − *ki* [B_T_] [P] (8)

d[L]/dt = *μ_L_* [L] (1 − [B_T_]/K_L_) + *f*_1_ *δ* [B_1_] + *f*_>1_ *δ* [B_>1_] (9)

d[D]/dt = *d* (1 − *f*_1_) *δ* [B_1_] + *d* (1 − *f*_>1_) *δ* [B_>1_] (10)

In theory, this model can be readily expanded to specify bacteria infected with any number of phage, e.g., B_2_, B_3_ [27], but we found that specifying B_0_, B_1_ and B_>1_ was sufficient.

This model was able to give good fits to the CII expression time-courses with all parameter values except for the FOLs (*f*_1_ and *f*_>1_) applied globally between the different IPTG concentrations (Figure 4B). Very low *f*_1_ values were obtained up to 10 µM IPTG with *f*_1_ ~0.24 at 20 µM IPTG. Estimated values for *f*_>1_ increased steadily from ~0.02 to 1 as the concentration of CII was increased, confirming CII’s critical role in setting the FOL. A striking feature of the optimal estimates is that *f*_>1_ > *f*_1_ for each IPTG concentration, suggesting that the phage 186 lysis-lysogeny decision is indeed responsive to the MOI.

To better appreciate the ranges of *f*_1_ and *f*_>1_ that are compatible with the combined CII expression time-course data, we systematically scored the fit between model and data over *f*_1_, *f*_>1_ space. The resulting heatmaps (Figure 4C; low scores indicate better fits) show that while there is considerable uncertainty in the *f*_1_ and *f*_>1_ estimates, that there is a clear increase in these FOLs with increasing CII expression and that *f*_>1_ > *f*_1_ holds for the preferred fits once IPTG exceeds 5 µM.

### 2.4. Application of the Multiple-Infection Model to the Phage 186 wt Time-Course

**Figure 5:**
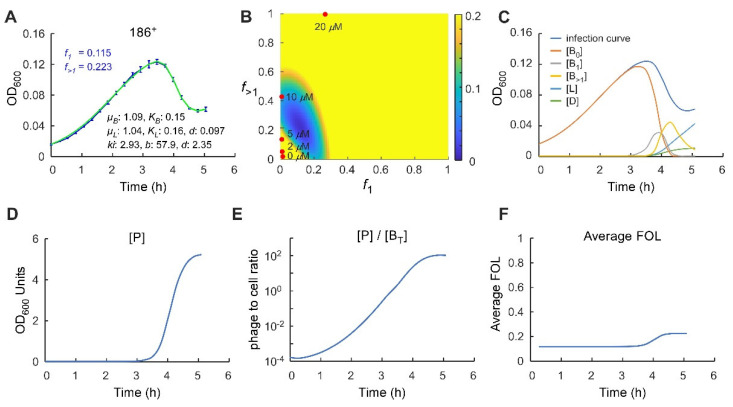
Analysis of 186^+^ phage infection time course using the multiple-infection lysogenisation model. (**A**) Fitting of the 186^+^ phage infection time course using the multiple-infection lysogenisation model. Data are taken from Figure 2A. Error bars represent standard deviation, *n* = 6. For data fitting, three rounds of 50,000 iterations of fitting were performed, and the best fit from each round was plotted. (**B**) Heatmaps showing the range of *f*_1_ and *f*_>1_ values that produce a good fit (blue). The best fit *f*_1_, *f*_>1_ values for 186 *cII*^−^ phage infection at each IPTG concentrations are overlayed. None of the fixed CII concentration precisely mimic CII expression in a phage 186^+^ infection. However, there are some overlap in *f*_1_, *f*_>1_ ranges between 10 µM IPTG and 186^+^ infection. (**C**–**E**) The concentrations of different species, the relative phage to cell ratio, and (**F**) the average *f* over the course of the 186^+^ infection as predicted by the multiple-infection lysogenisation model.

We next asked whether these features were also apparent in a more natural infection scenario, infection by 186^+^ of the non-CII-expressing host, where CII is made by the infecting phage. The multiple-infection model gives an excellent fit to the 186^+^ infection time course, giving optimal estimates for *f*_1_ and *f*_>1_ of ~0.11 and 0.22, respectively (Figure 5A). The result for *f*_1_ is consistent with previous 186 FOL measurements of ~10% at low MOAs [17] and the value for *f*_>1_ represents a mild MOI sensitivity. Scanning of *f*_1_, *f*_>1_ space showed that *f*_>1_ > *f*_1_ for the majority of good scores, supporting the idea that phage 186^+^ is MOI sensitive, however good scores can also be obtained with *f*_1_ = *f*_>1_ (Figure 5B). Interestingly, the 186^+^ *f*_1_ and *f*_>1_ estimates do not align well with any of the optimal *f*_1_, *f*_>1_ combinations for 186 *cII*^−^ infection of the CII expression strain (Figure 5B), suggesting that a fixed CII concentration does not precisely mimic CII expression in a phage 186^+^ infection. However, there are some *f*_1_, *f*_>1_ values that give good scores for both the 10 µM CII expression data and the 186^+^ data (Figure 4C and Figure 5B).

In the multiple-infection model, the parameter delta (*δ*) represents the timing between infection and cell lysis or lysogenisation. Estimates for delta cluster tightly around its optimal value of 2.4 h^−1^ (Figure S2), equating to an average time of 25 min, which conforms reasonably well to observed latent periods for phage 186 of ~30–40 min in single-step infection experiments [26,28]. The presence of delta frees up *k_i_* so that it can represent a simple rate of phage infection. The optimal estimate for *k_i_* of 2.9 OD_600_^−1^ h^−1^ implies that at a bacterial concentration of 1 OD_600_ unit under these conditions, a single phage would take ~20 min to infect. While this may seem slow, phage 186 infection is reasonably inefficient, and we routinely concentrate late log phase bacterial cultures five- to tenfold for infection experiments. The parameter *b* now should better represent the phage burst size, the number of phage produced per lytic cell, and the optimal estimate of *b* ~60 also accords well with observations for phage 186 [29]. As for the simple infection model, the estimates for *k_i_* and *b* tend to be anticorrelated (Figure S2).

We used the model to follow how the concentrations of different species are predicted to change over the course of the 186^+^ infection (Figure 5C–E). Strikingly, phage numbers remain very low until just 30 min before the OD_600_ peak and increase rapidly over the next 60 min (Figure 5D), equalling the number of bacteria at ~10 min before the peak, and reaching double the number of bacteria at the peak. The majority of infections, single and multiple, as well as lysogenisations occur in the period 30 min before and 30 min after the OD_600_ peak (Figure 5C). The maximal phage concentration at ~5 OD_600_ units (Figure 5D) is ~30-fold the bacterial concentration at carrying capacity (0.16 OD_600_ units), which is equivalent to ~5 × 10^9^ phage/mL, given that 0.16 OD_600_ units is ~1.6 × 10^8^ cells/mL. Such titres are comparable to those achieved in single-step phage infections [30,31]. The increasing phage concentrations cause the average FOL to shift from *f*_1_ to *f*_>1_ over this 60 min postpeak (Figure 5F).

## 3. Discussion

### 3.1. Control of the Phage 186 Lysis-Lysogeny Decision by CII

It has long been known that the CII protein of phage 186 is essential for the establishment of the lysogenic life cycle [17,21]. However, these studies indicated a digital ‘all-or-nothing’ response to changes in CII activity; an FOL close to 0% for cII-deficient phage or an FOL close to 100% for a stabilised CII mutant [23]. In agreement with this digital response, we showed here that even a moderate defect in CII activity, a mutation at *pE* that reduces its activity by 40% at high CII levels [18], caused an almost complete loss of lysogenisation. However, a graded response to CII levels was seen in the infection time-course analysis for 186 *cII*^−^ phage exposed to different levels of CII expressed by IPTG induction, with estimated FOLs ranging from ~1% to 27% for single infections and from 2% through 5%, 15%, and 43% to 100% for multiple infections (Figure 4). This result is the first demonstration that the lysogenisation frequency of phage 186 can be fine-tuned by regulating the expression levels of CII, and supports the idea that CII is a key regulator in the lysis-lysogeny decision. Stochastic modelling indicates that the instability of 186 CII confers a more rapid decision [17], a feature also proposed to be provided by instability of λ CII [25,32]. 186 CII is likely to also be a target for environmental signals that affect the decision, given it is degraded by both FtsH and RseP proteases [17].

### 3.2. Response to the Multiplicity of Infection

Analysis of the phage 186^+^ infection time-course with a multiple infection model that allows different FOLs for cells infected by a single or multiple phage suggested an approximate doubling of the FOL for multiple infections. This MOI response was more pronounced with infection of cells expressing CII by 186 *cII*^−^, possibly only because of the increased statistical power of the larger data set, but possibly because higher or fixed CII levels somehow increase MOI sensitivity. Such MOI sensitivity is proposed to allow temperate phage to sense the phage:bacteria ratio and to favour lysogeny when sensitive hosts are scarce. In λ, measurements of MOI sensitivity vary depending on experimental conditions. Estimates of *f*_1_ close to zero and *f*_2_ close to 1 have been obtained in bulk studies [11,12,33], while single-cell studies have given estimates of *f*_1_ ~30% and *f*_2_ ~50%, increasing to ~70% at *f*_5_ [34].

Lambda’s MOI response is thought to result from an increased chance of reaching a lysogenic threshold for CII when multiple phage genomes are present [7,8,33], in part due to titration of cellular proteases active against CII, and their inhibition by λ CIII [6,9,35]. Phage 186 is not known to encode a CIII-like function, but the sensitivity of 186 CII to cellular proteases [17] could result in multiple infections giving an increased level of CII relative to other phage proteins if these proteases become overwhelmed. However, this does not explain the presence of the MOI effect in the CII-expressing cells infected by 186 *cII*^−^, where the level of CII does not change with MOI and would presumably be *lower* relative to other phage proteins at higher MOIs. Thus the phage 186 MOI effect may be achieved differently from λ. It is also possible that, while our MOI-sensitive model can explain the data, 186 may actually be using some other signal to increase lysogenisation around the OD_600_ peak. For example, phage SPbeta uses a ‘phage quorum’ chemical produced by previous infections to increase lysogenisation as phage:bacteria ratios increase [33,36].

## 4. Materials and Methods

### 4.1. Strains

*Escherichia coli (E. coli)* strain BW25113 [Δ*(araD-araB)567,* Δ*lacZ4787(::rrnB-3), λ-, rph-1,* Δ*(rhaD-rhaB)568, hsdR514*] [35] was used as a general host strain, unless otherwise stated. Strains AH1839 and IM514 are both derivatives of BW25113 that carry either wt or *cII*^−^ (helix-turn-helix mutant) 186 prophage [17]. The 186 *pE_down* strain KS54 is derived from *E. coli* C600 [*thr-1, leuB6(Am), fhuA21, cyn-101, lacY1, glnX44(AS), λ-, e14-, rfbC1, glpR200(glpc), thiE1*] and is a lysogen of 186 that carries an A to G mutation at 186 *pE* promoter that reduces CII mediated *pE* activation [18].

### 4.2. Phage

The 186 *cII*^−^ and 186 *pE* mutant phage were produced from IM514 and KS54, respectively. The 186 *cI10* is a clear plaque mutant, which has a defective *cI* gene due to a frameshift mutation [21].

### 4.3. Plasmids

The pZS45-cII169 is a low copy number vector for expression of wide type cII under control of Isopropyl β-d-1-thiogalactopyranoside (IPTG) inducible *plac* promoter [23]. The pUHA-1 plasmid (a gift from H. Bujard, Heidelberg University, Heidelberg, Germany) was used to supply a constant level of LacI repressor.

### 4.4. Phage Titring and MOA Calculation

Indicator strain (BW25113) was grown in lysogeny broth (LB) until OD_600_ ~0.5. 300 μL of indicator cells was then mixed with 10 μL of each of eight 1:10 serial dilutions of the phage stock in TM (10 mM Tris-HCl, pH 7.5, 10 mM MgSO_4_) buffer and 3 mL of melted (48 °C) soft agar (0.7% *w*/*v*) supplemented with 10 mM MgSO_4_ and 5 mM CaCl_2_. The mixture was quickly but very gently poured onto pre-warmed LB agar plates and rotated gently to evenly distribute the mixture. The plate was left at room temperature for 15 min for the top layer to set before moving to 37 °C for overnight incubation. Plates exhibiting ~100 well-isolated plaques were used to calculate the phage titre. A plate stock of 186^+^ typically gives ~1 × 10^9^ plaque forming units (pfu)/mL, and a plate stock of 186 *cI10* typically gives ~1 × 10^10^ pfu/mL. MOA is calculated as the number of phage (pfu) added at the beginning of infection over the number of cells to be infected.

### 4.5. Microtitre Plate-Based Phage Infection Assay

Cells were initially streaked onto a 1.5% LB agar plate, and an individual bacterial colony was then picked to inoculate an overnight culture in Tryptone Broth (1% Oxoid™ Tryptone and 86 mM NaCl) supplemented with 5 mM CaCl_2_ (TBC). The overnight culture was diluted 100-fold in TBC and then grown to OD_600_ ~0.4 (~2.5 h at 37 °C). For cells carrying the pZS45-cII169 and pUHA-1 plasmids, spectinomycin (50 µg/mL), kanamycin (50 µg/mL), and IPTG (0–20 µM) were also added. The culture was then diluted in TBC to OD_600_ 0.05, and 185 µL of culture was transferred to each well of a 96-well microtitre plate together with 15 µL of diluted phage stock at MOA of 1.5 × 10^−4^ or TM buffer alone as a no phage control. The infection time course was monitored by incubating the microtitre plate at 37 °C in a Victor X5 plate reader (Perkin-Elmer) equipped with a 600 nm optical filter (Perkin Elmer, 600/8 nm, 1420–521). The injector accessory was used to add 5 μL of sterile water per well every 15 min to compensate for volume loss due to evaporation, as per Maynard et al. [20]. Eight replicates for each condition were assayed, but for data analysis, only the results obtained from the inside six wells were included as evaporation is generally higher for outside wells than inside wells.

### 4.6. Fitting Procedure

Data were imported into MATLAB for processing. The OD_600_ measurements were background subtracted (0.036 for TBC broth) and stored as a series of 12 × 8 matrices, each of which corresponds to one time-point. The time at which the first measurement was taken was defined as time 0.

Simulations were performed by numerically integrating a given set of parameters with a MATLAB built-in ordinary differential equation solver (ode45) using Equations (1)–(4), modified from Maynard et al. [20] or Equations (5)–(10), modified from Sinha et al. [27]. The initial OD_600_ value was taken as the initial concentration of bacteria B_0_. The initial concentrations of infected cells ([B_1_] and [B_>1_]), lysogens [L] and cell debris [D] were set to zero, and the initial concentration of phage [P] was calculated as the product of [B_0_] and MOA.

Model fitting was performed with a Monte Carlo simulated annealing approach. The expected OD_600_ values were calculated at each of the experimentally tested timepoints with an initial guess of parameters. The simulated values were compared with the experimentally observed OD_600_ values by calculating a score = Σ{(measured OD − simulated OD)^2^/simulated OD}. Since each condition was repeated 6 times, the overall score was computed as a sum of all six individual scores calculated from each of the experimental repeats. The fitted parameter values (*ki*, *b*, *δ*, and *f*) were then varied at random by a factor of 0.9–1.1, and a new score was calculated using this new parameter set. If the new score was better than the previous score, the new parameter set was retained, and the score was updated, otherwise the new parameter set was rejected. To reduce the complexity of the fitting, the growth parameters for nonlysogen and lysogen (*µ_B_*, *K_B_*, *µ_L_*, and *K_L_*) were fixed from the growth curve fit. The cell debris coefficient *d* was determined using data obtained from lytic only 186 *cI10* phage infections. The model assumes that adsorption of phage to the cell debris is negligible. The best fit value of *d* was determined to be 0.1.

To reduce the search time, the parameters were allowed to vary only within a range deemed appropriate. The boundaries of the *ki* and *b* were set to be within the range 1.2 to 3, and 20 to 200, respectively, while *f* may fall anywhere between 0 and 1. A typical run involved three rounds of 50,000 iterations of fitting, which took ~5 min on a MacBook Pro.

## 5. Conclusions

Infection time-courses provide a simple and relatively high-throughput method to examine interactions between lytic phage and their bacterial hosts in a more ecologically relevant way than traditional single-step growth experiments. We have shown that analysis by an improved mathematical model can extract key infection parameters from a single time course. Together, these approaches can be used to examine effects of phage and host mutants and can be easily applied to provide useful information about less well-characterised phage and hosts of ecological or medical importance.

## Data Availability

Data is contained within the article and Supplementary Material.

## Supplementary Materials

The following are available online at https://www.mdpi.com/article/10.3390/ph14100998/s1, Figure S1: Control growth and infection curves. Figure S2: Analysis of fitting parameters.
