# Supplementary figures and images for: Analysis of Infection Time Courses Shows CII Levels Determine the Frequency of Lysogeny in Phage 186
Source: Pharmaceuticals (Basel). 2021 Sep 29;14(10):998. doi: 10.3390/ph14100998 (PMC8538670; doi:10.3390/ph14100998)

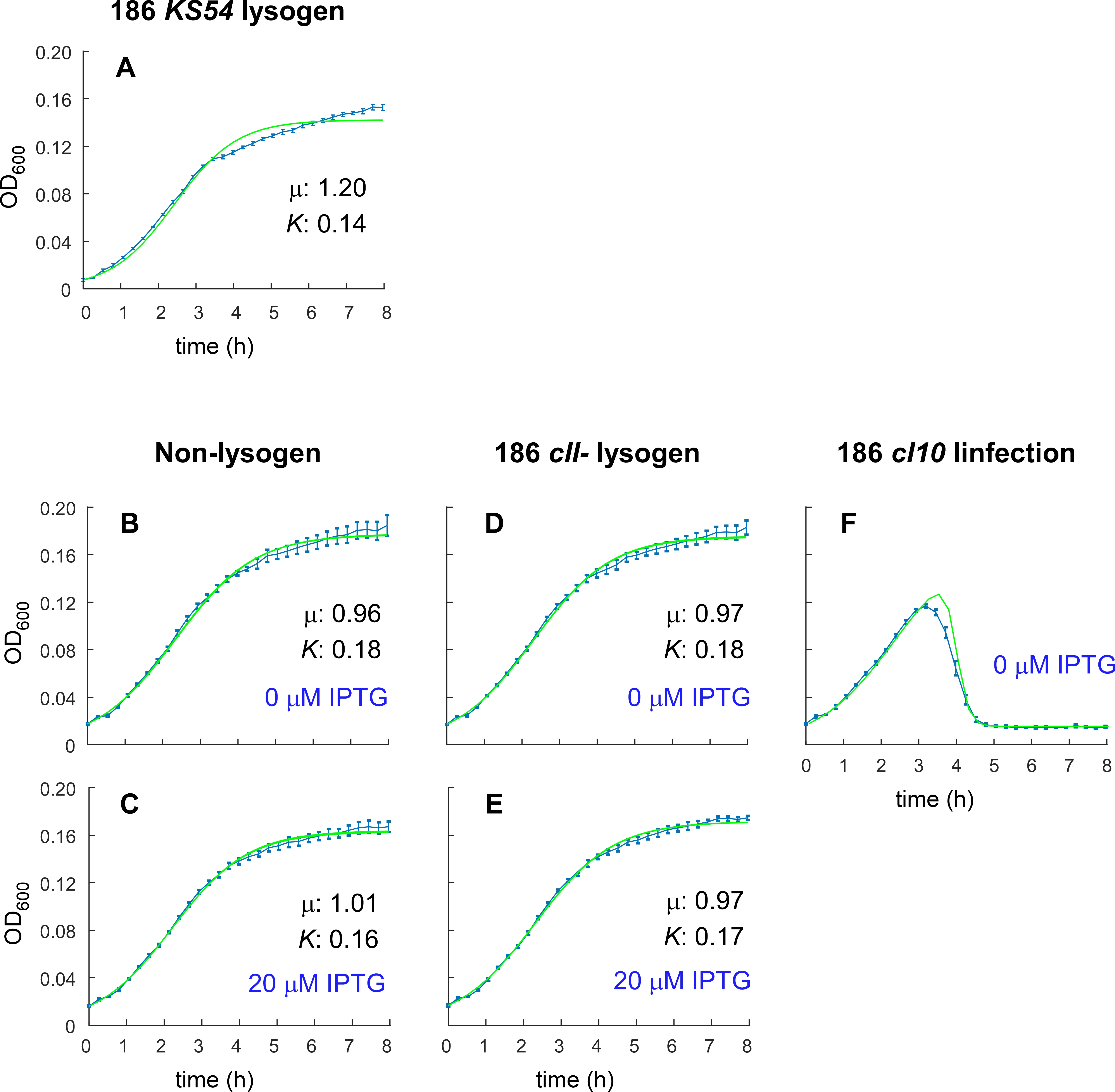

Supplement: Supplementary file 1 [file pharmaceuticals-14-00998-s001.zip › Fig S1 proofs.jpg]

**A**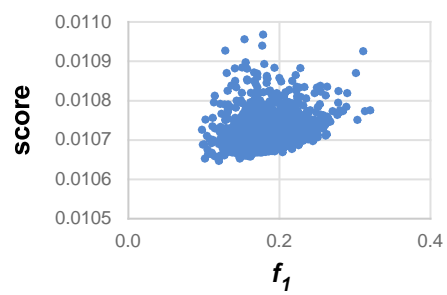**B**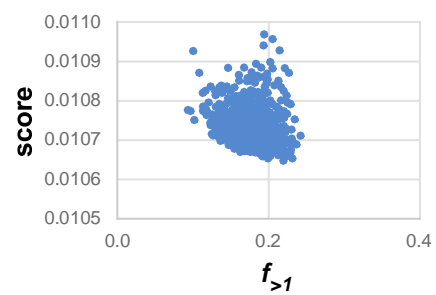**C**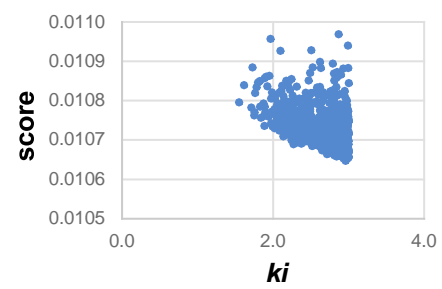**D**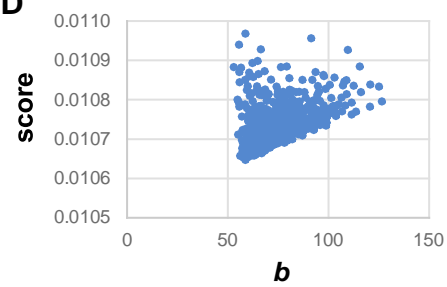**E**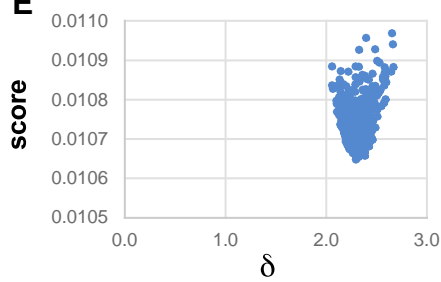**F**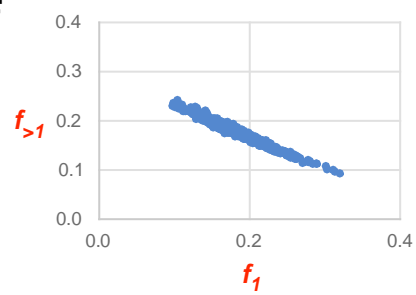**G**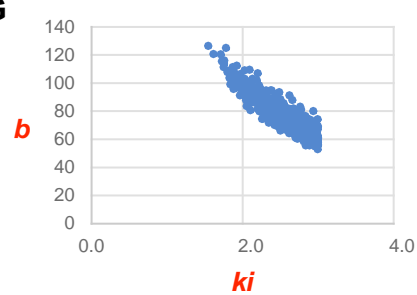

Supplement: Supplementary file 1 [file pharmaceuticals-14-00998-s001.zip › Fig S2.pdf]
